# Examining How Cognition, Neighborhood Characteristics, and Physical Function are Associated with Outdoor Frequency Among United States Community‐Dwelling Medicare Beneficiaries

**DOI:** 10.1002/alz.093346

**Published:** 2025-01-09

**Authors:** Justine S. Sefcik, Martha C Coates, Darina Petrovsky, Amy Glasofer, Safiyyah Okoye, Daniel T Vader, Renee H Moore, Zachary G Baker, Kris Pui Kwan Ma, Zahra Rahemi, Juanita‐Dawne R Bacsu, Matthew Lee Smith, Rose Ann DiMaria‐Ghalili

**Affiliations:** ^1^ Drexel University College of Nursing and Health Professions, Philadelphia, PA USA; ^2^ Duke University, Durham, NC USA; ^3^ Drexel University, Philadelphia, PA USA; ^4^ Drexel University Dornsife School of Public Health, Philadelphia, PA USA; ^5^ Arizona State University, Phoenix, AZ USA; ^6^ University of Washington, Seattle, WA USA; ^7^ Clemson University, Greenville, SC USA; ^8^ Thompson Rivers University School of Nursing, Kamloops, BC Canada; ^9^ Texas A&M University, College Station, TX USA

## Abstract

**Background:**

A large body of research supports the benefits of older adults engaging in physical activity outdoors. However, a paucity of research explores factors associated with the frequency of older adults going outdoors. The aim of this study was to explore how factors including cognition, neighborhood characteristics, and physical ability were associated with community‐dwelling older adults’ outdoor frequency.

**Method:**

This cross‐sectional study used National Health and Aging Trends Study data to investigate determinants of outdoor frequency among Medicare beneficiaries aged 65 and older. We descriptively characterized the distribution of outdoor frequency by participant demographic, health, and neighborhood characteristics using counts and percentages, and estimated odds ratios of relationships between key characteristics (including cognitive status, neighborhood characteristics, and physical ability) and outdoor frequency using survey ordinal logistic regression.

**Result:**

The final sample included 3,368 participants, ages 65 and older (57% female). Most participants went outside every day (60.4%) and few went outside rarely or never (3.5%). Compared to those who went outside daily, individuals who went outside rarely or never were more likely to have probable dementia, live in neighborhoods with street disorder and continuous sidewalks, and have physical limitations requiring assistive devices and help to go outside.

**Conclusion:**

Older adults with dementia and physical limitations may be less likely to experience the benefits of outdoor activity. Neighborhood characteristics may contribute to going outdoors. More research is needed to understand the experiences of people living with dementia and physical limitations in order to develop interventions and age‐friendly neighborhoods promoting outdoor activity.

